# pH-Responsive, Thermo-Resistant Poly(Acrylic Acid)-g-Poly(boc-L-Lysine) Hydrogel with Shear-Induced Injectability

**DOI:** 10.3390/gels8120817

**Published:** 2022-12-12

**Authors:** Maria-Eleni Karga, Maria-Eleni Kargaki, Hermis Iatrou, Constantinos Tsitsilianis

**Affiliations:** 1 Department of Chemical Engineering, University of Patras, 26500 Patras, Greece; 2 Department of Chemistry, University of Athens, Panepistimiopolis, Zografou, 15771 Athens, Greece

**Keywords:** hydrogel, poly(acrylic acid)-g-poly(boc-l-lysine), hydrophobic association, frozen network, shear-induced injectability, self-healing, pH responsiveness

## Abstract

In this study we report the rheological behavior of aqueous solutions of an amphiphilic graft copolymer constituting a polyacrylic acid (PAA) grafted by poly(boc-L-lysine), P(b-LL). Due to the highly hydrophobic nature of the grafted chains, the copolymer self-assembles spontaneously in aqueous media forming three-dimensional (3D) finite size networks (microgels). The rheological analysis demonstrated that the copolymer behaves as a strong elastic hydrogel, showing characteristics of a “frozen” network. Moreover, it is noteworthy that the formulation shows the above-described characteristics in very small concentrations (0.25–1.20 wt%) compared to other naturally cross-linked hydrogels that have been studied so far. Concentration significantly affects the rheological properties of the hydrogel, showing considerable increase in elastic modulus, following the scaling law G’~C^1.93^. At the same time, the hydrogels can be described as intelligent stimuli-responsive systems, showing pH and shear responsiveness as well as stability with temperature changes. Thanks to the pH dependance of the degree of ionization of the weak polyelectrolyte PAA backbone, stiffness and swelling of the hydrogels can be tuned effectively by adjusting the pH conditions. Simulating conditions such as those of injection through a 28-gauge syringe needle, the gel demonstrates excellent response to shear, due to its remarkable shear thinning behavior. The combination of pH-sensitivity and shear responsiveness leads to excellent injectability and self-healing properties, given that it flows easily upon applying a low stress and recovers instantly in the site of injection. Therefore, the physically cross-linked PAA-g-P(b-LL) hydrogel exhibits remarkable features, namely biocompatibility, biodegradability of cross-links, pH responsiveness, shear-induced injectability and instantaneous self-healing, making it a potential candidate for various biomedical applications.

## 1. Introduction

Self-assembling hydrogels represent a very interesting class of soft matter attracting great attention during the last decades, thanks to their wide range of (potential) applications, some of which concern biomedical applications, namely tissue engineering, controlled drug delivery, etc. [1,2,3]. Self-assembling hydrogels relies on a three-dimensional (3D) non-permanent network, created through the self-organization of associative macromolecules (gelators) of special architecture and functionality in aqueous media [4,5]. The simplest gelator comprises a long hydrophilic block carrying a number of short hydrophobic blocks (also named stickers) which trigger intermolecular hydrophobic association leading to a 3D network.

When the hydrophilic block is a weak polyelectrolyte, either anionic or cationic, the degree of ionization of the macromolecule, which strongly affects the chain conformation (from coil to rigid), depends on the solution pH and the pK_a_ of the ionogenic functionality of the repeating monomer units [6]. Moreover, the functional groups (e.g., COOH) are susceptible to easy modification reactions (e.g., amidation), yielding the so-called hydrophobically modified polyelectrolytes, which are effective gelators in aqueous solutions. Providing that the ionization (charge density) of the gelator affects the network connectivity and hence the rheological properties of the hydrogel [7], pH can be used as stimulus, rendering the hydrogel a pH-responsive system [8,9].

Attention must also be paid to the hydrophobic strength of the stickers that form, after association, the micellar type of physical cross-links. If the exchange of the hydrophobic blocks between the cross-links is relatively rapid (low relaxation times within the experimental time) the system behaves dynamically and can attain equilibrium. On the contrary, for long relaxation times (practically immeasurable), kinetically “frozen” hydrogels are formed [10].

Associative poly(acrylic acid) (A block) based gelators bearing hydrophobic stickers in various macromolecular architectures, namely BAB telechelic [11] and BAC heterotelechelic polyelectrolytes [12]; (AB)_n_ star block [13] and (A-g-B) graft copolymers [14], have been designed and explored as gelators previously. The graft copolymer architecture, although not very well-defined due to the random distribution of the pendant stickers, is more versatile from a synthetic point of view (easy grafting reactions) and more appealing to applications.

This paper is devoted to the development of self-assembling hydrogels formed by the association of PAA, grafted by poly(boc-L-lysine) (P(b-LL)), which acts as a hydrophobic sticker. The main novelty of this work is the use biodegradable and/or transformable polypeptide side chains as stickers. The hydrophobic P(b-LL), bearing boc-protected L-Lysine residues, is easily hydrolysable in strongly acidic media, transforming the copolymer to PAA-g-PLL, which in fact is a weak polyampholyte that is expected to be self-organized through ionic interactions [15]. The prepared hydrogel exhibits pH sensitivity, shear-induced injectability and self-healing, while it is stable upon heating. All these properties seem to make the PAA-g-P(b-LL) hydrogel innovative, constituting it as a good candidate for biomedical applications.

## 2. Results and Discussion

### 2.1. Gelation and Polymer Concentration Dependence

**Figure 1:**
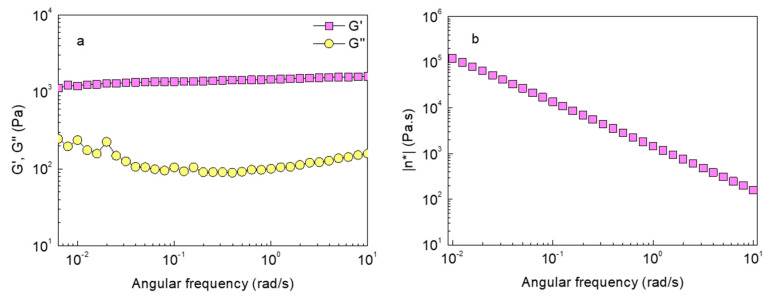
Angular frequency dependence of (**a**) storage (G’) and loss (G”) modulus and (**b**) complex viscosity |η*|, of PAA-g-P(b-LL) aqueous solution of, C_p_ = 1 wt%, at pH 8.8.

**Figure 2:**
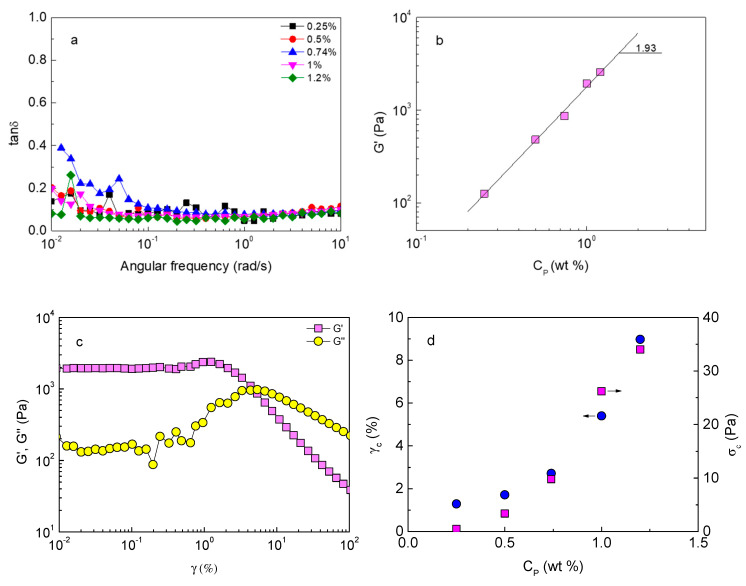
(**a**) Angular frequency dependence of tanδ at various concentrations, (**b**) G’ at 1 Hz obtained from the strain sweep data, (**c**) strain sweep of C_p_ = 1 wt% sample at 1 Hz and (**d**) γ_c_, σ_c_ versus C_p_ of PAA-g-P(b-LL) in aqueous solutions at pH 8.8.

The PAA-g-P(b-LL) graft copolymer self-assembles spontaneously in aqueous media due to the association of the hydrophobic P(b-LL) sticky side chains, forming highly viscous solutions. Initially, an aqueous solution of 1 wt% polymer concentration was prepared, the pH of which was measured to be 8.8, implying neutralization of the carboxy units of the PAA backbone. To study their rheological properties, oscillatory shear experiments were conducted in the linear viscoelastic regime defined by strain sweep measurements (Figure S2). Figure 1 demonstrates the frequency dependence of the storage (G’) and loss (G”) modulus, along with the complex viscosity |η*|. As shown, G’ is higher than G” and both moduli are weakly dependent on frequency. The terminal relaxation zone is not visible in the low frequencies which implies nearly immeasurable relaxation times. This is also reflected in the absence of any plateau in the complex viscosity versus frequency plot (Figure 1b). Additionally, the loss tangent (tanδ = G”/G’) is of the order of 0.1 in the whole frequency range investigated (Figure 2a). These data clearly show gel-like behavior which agrees with the observed formation of a free-standing gel and resembles the behavior of chemically (irreversible) cross-linked systems. This behavior is attributed to the formation of a 3D network formed by intermolecular hydrophobic physical cross-linking of the pendant P(b-LL) chains bridging the copolymer chains. Provided that the P(b-LL) chains are highly hydrophobic, the exchange dynamics of the P(b-LL) from the hydrophobic junctions cannot occur. Thus, a kinetically frozen system is forming, the structure of which depends on the preparation history. As we have observed during the preparation step (see Section 2.4, Figure 11), the aqueous formulations should be constituted of a suspension of densely packed swollen microgels (finite size 3D networks) rather than an infinite spanning network.

To evaluate the effect of concentration on the viscoelastic response of the PAA-g-P(b-LL) hydrogel, the 1 wt% solution was diluted successively forming various concentrations down to 0.25 wt%, while a new 1.2 wt% solution was prepared at the same pH of 8.8. In all concentrations investigated, G’ exceeds G” along the entire frequency range and G’ are nearly frequency independent. However, the loss tangent values are of the order of 0.1, at least for ω > 0.1, as seen in Figure 2a. Hence, the solutions behave as elastic, soft solids even at the lowest C_P_ (0.25 wt%) investigated. The storage modulus, G’, reflects the connectivity of the network in terms of degree of jamming, i.e., whether the swollen microgels are more or less closely packed. Note that even a 5 wt% aqueous solution of the PAA precursor does not exhibit any elasticity (Figure S3 in Supplementary Materials), which confirms that the elastic behavior of the graft copolymer is solely due to the physical cross-linking induced by the hydrophobic association of the P(b-LL) grafts forming microgels. In Figure 2b, G’ (1 Hz) is plotted as a function of C_P_. As seen, the storage modulus increases with polymer concentration following the scaling law of G’~ C_P_^1.93^. The storage modulus augmentation with the gelator concentration should be attributed to the increase in the number density of the microgels in the media which leads to an increasing network connectivity.

Oscillatory strain sweeps at a constant frequency of 1 Hz were also conducted beyond the linear regime in all samples of different polymer concentrations at pH 8.8. A characteristic example is demonstrated in Figure 2c for the 1 wt% formulation. The strain response of the hydrogels reveals interesting hydrogel characteristics. Firstly, the elastic response of the formulations (G’ > G”) is dominant at low stresses and secondly, the strain threshold (γ_LVR_) denoting the linear viscoelastic regime (G’ independent of strain) is very low (i.e., <1%) for all the examined samples. The same behavior has been reported for other associative polymers bearing highly hydrophobic stickers, forming kinetically “frozen” hydrogel systems [11,16,17,18]. Surprisingly, a strain hardening effect can be observed (G’ increase) which might indicate an increasing network connectivity prior the shear thinning at elevated strains. This behavior was not observed in the formulations of C_P_ < 1 wt%. Furthermore, a well-defined critical strain amplitude, γ_c_, can be determined as the strain corresponding to the crossover point (G’ = G”), beyond which the formulation flows (G” predominates G’), reflecting substantial structural rearrangements in the system. As demonstrated in Figure 2d, γ_c_ increased with polymer concentration, implying that the increase in the number density of the swollen microgels causes an increase in the critical strain amplitude, σ_c_, needed to break the structure that resists the hydrogel flow. Indeed, in the right axis of Figure 2d we can see the increasing corresponding oscillatory stress, σ_c_, with C_P._ For the highest C_P_ of 1.2 wt%, the critical stress and the corresponding generated critical strain are 34 Pa and 8.98%, respectively, showing that above a relatively slow stress and strain, the hydrogel easily flows. This is consistent with the assumption that the structure is constituted of a suspension of densely packed swollen microgels. Similar behavior, i.e., low critical stress, has been observed for hydrogels formed by swollen, chemically cross-linked PAA microgels (Carbopol) [19]. Likewise, the increase of γ_c_, reflecting the extension of LVR, with C_P_ is consistent with the Carbopol behavior [20], which corroborates the assumption of a closely packed microgel structure for the present system.

### 2.2. Temperature Effect

**Figure 3:**
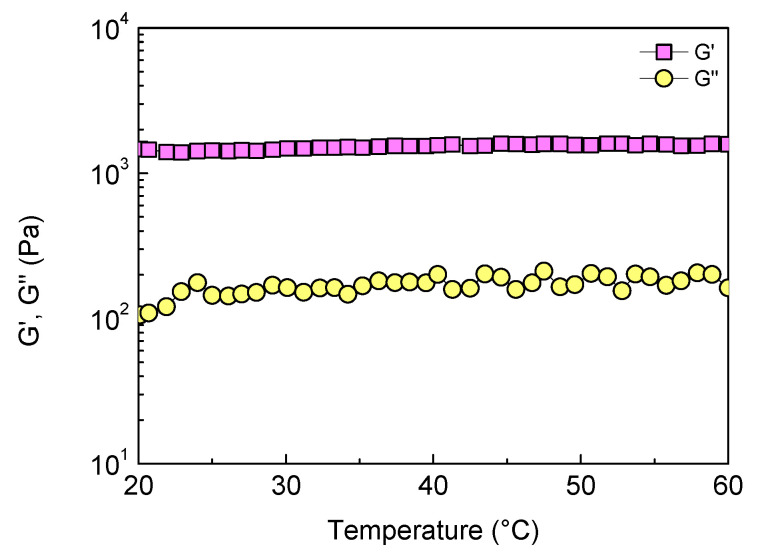
Storage (G’) and loss (G”) modulus as a function of Temperature (1 °C/min) of a PAA-g-P(boc-LL) aqueous solution of C_p_ (1 wt%) at pH 8.8.

The temperature dependence of the viscoelastic response of the hydrogel is an important parameter also relevant to the hydrogel performance. Figure 3 demonstrates G’ and G” as a function of temperature, obtained by a temperature ramp experiment performed on the 1 wt% sample with a heating rate of 1 °C/min at a constant frequency of 1 Hz. The moduli remain nearly constant upon heating, revealing a remarkable resistance to heat at least up to 60 °C. Similar behavior has been reported for associative polyelectrolytes of triblock topology with hydrophobic end-blocks [16,17] and (AB)_4_ tetra-armed stars with relatively long hydrophobic outer blocks B [18]. This behavior implies that the formed network structure in the hydrogel remains intact upon heating. This is attributed to the lack of exchange dynamics of the stickers when they are adequately long and hence highly hydrophobic, as they form stable junctions (physical cross-links) that resist the enhanced thermal molecular motion. Indeed, in our case, the P(b-LL) stickers are long (the degree of polymerization is 50) and highly hydrophobic forming “frozen” junctions by the hydrophobic association. A practical consequence of this behavior is that the rheological properties will not alter from room temperature (20–25 °C) to physiological temperature (37 °C) which might be beneficial for bioapplications.

### 2.3. pH Responsiveness

**Figure 4:**
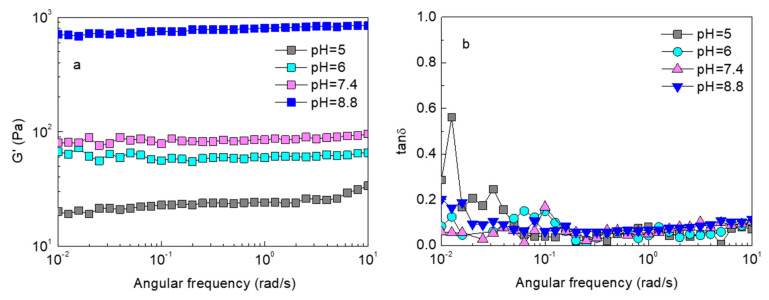
Angular frequency dependence of G’ (**a**) and tanδ (**b**) at various pHs of PAA-g-P(boc-LL) aqueous solutions of C_p_ = 0.5 wt%.

Hydrogels based on weak polyelectrolytes, the ionization of which depends on pH (and their pK_a_), have been reported to respond to pH changes [7,21,22,23,24,25]. Thus, pH as a stimulus is a very important factor, especially for biomedical applications, motivating the exploration of its influence on the hydrogel rheological properties. Keeping the polymer concentration constant at 0.5 wt%, a number of aqueous formulations of pH 5, 6, 7.4 and 8.8 were prepared and explored by oscillatory rheology within the linear viscoelastic regime, setting γ = 0.05 % and frequency = 1 Hz. The elastic modulus, G’, dominates over G” in the entire range of frequencies (10^−2–^10^1^ rad/s) tested in all cases. Additionally, G’ had little dependence on the frequency and the magnitude of the loss tangent, tanδ, which lies in the range of 0.2–0.1, as can be observed in Figure 4. These data show that pH, in the range investigated, does not influence the general behavior of an elastic “frozen” soft hydrogel, implying a percolating structure of swollen microgels in all cases.

**Figure 5:**
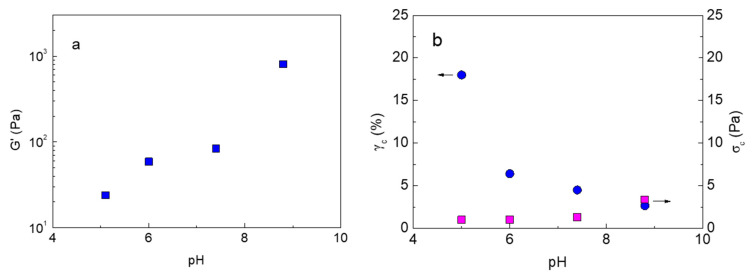
pH dependence of G’ (at 1 rad/s, from Figure 4a), (**a**) γ_c_ (spheres) and σ_c_, (squares, from strain sweep data) (**b**) of PAA-g-P(b-LL) aqueous solutions of C_p_ = 0.5 wt%.

However, significant differences were observed in the magnitude of the moduli. As indicated in Figure 5a, the storage modulus increases with pH by more than one order of magnitude, from 5 to 8.8, suggesting significant strengthening of the hydrogel. This remarkable pH dependence should be related to the degree of ionization, *α*, of the PAA backbone of the graft copolymer, which increases from 0.35 at pH 5 to 0.97 at pH 8.8 (nearly fully neutralized PAA) [26], which affects the PAA chain conformation (flexibility) and the microgel swollen ability. As *α* decreases on lowering the pH, the backbone chains (constituting the bridging chains of the network) become unstretched and flexible [25] and the microgel size decreases. Hence, the network connectivity is reduced, which is reflected in the diminishing of the elasticity of the hydrogel, as manifested by the variation in G’.

Interesting data concerning the pH dependence of the critical γ_c_ above which the gel flows are presented in Figure 5b. As observed, γ_c_ decreases about seven-fold from pH 5 to 8.8. At C_P_ = 0.5 wt %, the connectivity of the network is weak, although a percolated structure persists. This is reflected in the low critical σ_c_ needed to be applied for the hydrogel to flow (Figure 5b). Notably, σ_c_ varies smoothly with pH but the corresponding γ_c_ decreases remarkably upon increasing pH. Indeed, at pH 5 this low stress generates a much higher critical strain (about a seven-fold increase) with respect to pH 8.8. This effect is probably due to the higher flexibility of the PAA bridging chains and/or the higher deformability of the less swollen microgels at lower pH.

### 2.4. Shear Responsiveness and Self-Healing

**Figure 6:**
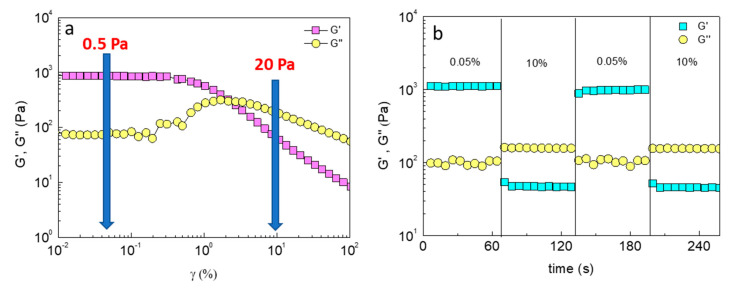
(**a**) G’ and G” as a function of strain at 1 Hz and (**b**) consecutive time sweep at fixed strain amplitudes as indicated by the arrows in (**a**), i.e., at 0.05% (LVR) and 10% (beyond LVR) of PAA-g-P(b-LL) aqueous solutions of C_p_ = 0.74 wt%.

Biomedical applications, enabling minimally invasive implantation in vivo through direct injection or catheter-based delivery as well as 3D/4D printing, requires hydrogels exhibiting effortless injectability and self-healing properties. To this end, the hydrogels in the present work were explored by suitably designed shear and oscillatory experiments to estimate these properties. The hydrogel was subjected to a consecutive stepwise time sweep under different strain amplitudes, dictated from the strain sweep data applied for the formulation of C_p_ = 0.74 wt% (pH 8.8). As demonstrated by the arrows in Figure 6a, oscillatory time sweeps were selected to perform repeatedly at stresses of 0.5 Pa and 20 Pa, which generated strain amplitudes of 0.05 and 10%, respectively, which is below the critical γ_c_ (LVR region) and well above the γ_c_ (shear-thinning region). Figure 6b illustrates the time dependence of G’ and G” with four steps of 60 s time intervals for each step. As shown, upon applying a strain in the LVR region, the formulation behaves as a strong hydrogel, since G’ dominates over G”, with an elastic modulus of 1100 Pa (tanδ = 0.095). Upon a sudden change in strain at 10 %, the formulation instantly flows as G” outweights G’ (tanδ = 3.34) with G’ dropping to 47 Pa. The hydrogel was instantly recovered in the third step where a low strain of 0.05 % was applied, as suggested by the values of G’ and tanδ (1000 Pa and 0.095, respectively). In the fourth step, the data are similar to those in the second step (the formulation flows) showing, in addition, excellent reversibility. The data in Figure 6b clearly indicate that the hydrogel exhibits self-healing properties, provided that it responds nearly instantly to abrupt variations in the applied shear stress with exceptionally fast hydrogel recovery following the hydrogel rupture induced at elevated stress.

**Figure 7:**
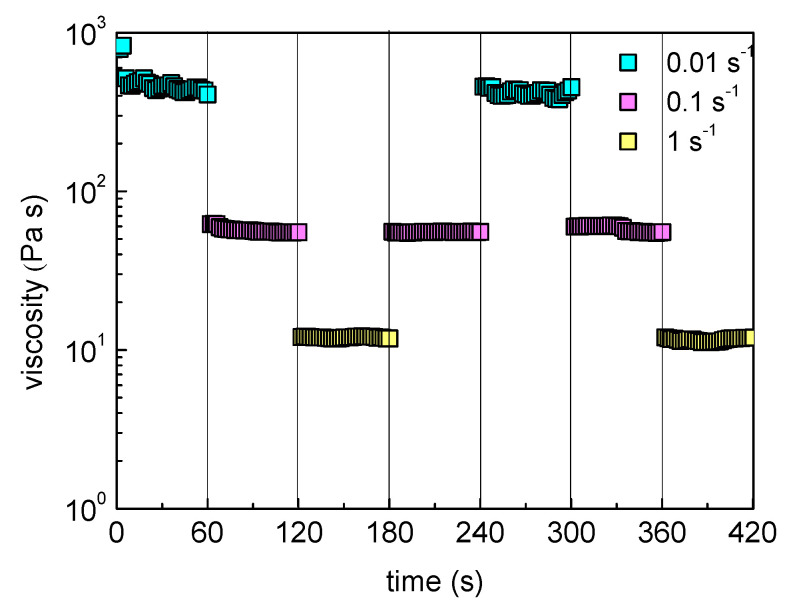
Consecutive shear viscosity time sweep in time intervals of 60 s for the 1 wt% PAA-g-P(boc-LL), pH 8.8 formulation, subjected to stepwise shear rate variations as indicated.

Hydrogel injectability, for applications in high gauge needles, relies on the shear thinning effect and requires low shear viscosities in the order of 1 Pa.s or less at elevated shear rates [27,28,29]. The shear responsiveness was first examined by steady state shear experiments applying different shear rates stepwise, at time intervals of 60 s. As can be observed in Figure 7, the apparent viscosity (*η*) decreases instantaneously with increasing shear rate in every step, confirming the shear thinning behavior of the hydrogel. Upon a decrease in shear rate, *η* is recovered instantly to the same values as in the previous steps, in agreement with the self-healing properties observed by oscillatory experiments (Figure 6b). Overall, the data in Figure 7 show excellent shear responsiveness of the hydrogel. The same behavior was observed for all concentrations.

**Figure 8:**
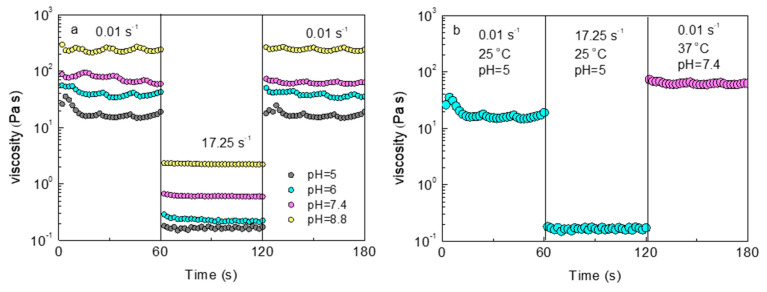
(**a**) Consecutive shear viscosity time sweep in time intervals of 60 s for the 0.5 wt% formulation at various pHs, as indicated. (**b**) Shear viscosity versus time in time intervals of 60 s, for the same formulation simulating an injection procedure.

To estimate the injectability through a syringe needle for formulations (e.g., C_P_ = 0.5 wt%) with various pHs, consecutive shear viscosity time-sweep stepwise experiments were conducted at room temperature (25 °C) and under shear rates of 0.01 and 17.25 s^−1^. The first, low value is close to the zero-shear viscosity (gel at rest) and the second value exemplifies the shear rate applied through a 28-gauge syringe needle inducing flow [30]. Figure 8a demonstrates the time sweep of the apparent viscosity at alternating shear rates, indicating excellent shear response of the hydrogel at all pHs explored. The viscosity drops by about two orders of magnitude upon applying a sudden variation in shear rate from 0.01 to 17.25 s^−1^ and recovers to the previous values when the shear rate is reverted back to 0.01 s^−1^. Notably, *η* decreased with decreasing pH in all steps in accordance with the oscillatory results depicted in Figure 4a. More importantly, at a shear rate of 17.25 s^−1^ (applied during injection), *η* drops to values lower than 1 Pa.s for pH ≤ 7.4, which meets the criteria for suitable injectability for the 0.5 wt % formulation. Taking advantage of the pH responsiveness, an appropriate injection procedure might be the following (Figure 8b): 

Preparation of the formulation at pH 5, exhibiting the lowest *η* of 0.17 Pa.s at 17.25 s^−1^ (easy flow) generated by applying just 3 Pa stress (painless injection). Upon relaxing the stress, the viscosity will abruptly rise by about three orders of magnitude under physiological conditions of pH 7.4 and 37 °C (gel recovery at the site of injection).

**Figure 9:**
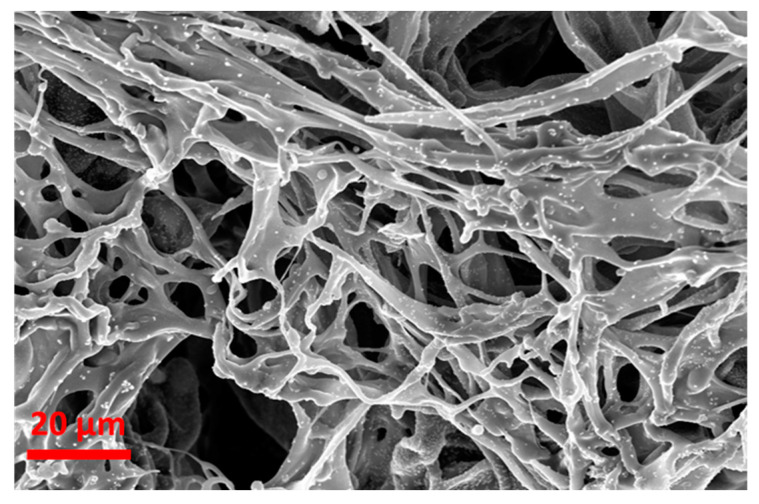
SEM micrograph of the hydrogel 3D structure after removing water of the sample (C_P_ = 0.5 wt%, pH 7.4) by freeze drying.

The morphology of the hydrogel (0.5 wt% formulation) was investigated by scanning electron microscopy after removing the water content by freeze-drying. Figure 9 (see also Figure S4 in Supplementary Materials) illustrates a characteristic image of the 3D structure, revealing an irregular, highly microporous material with mean pore size ranging from several μm to tens of μm, which is favorable for applications such as cell transplantation.

### 2.5. Hydrogel after Deprotection of P(b-LL)

**Figure 10:**
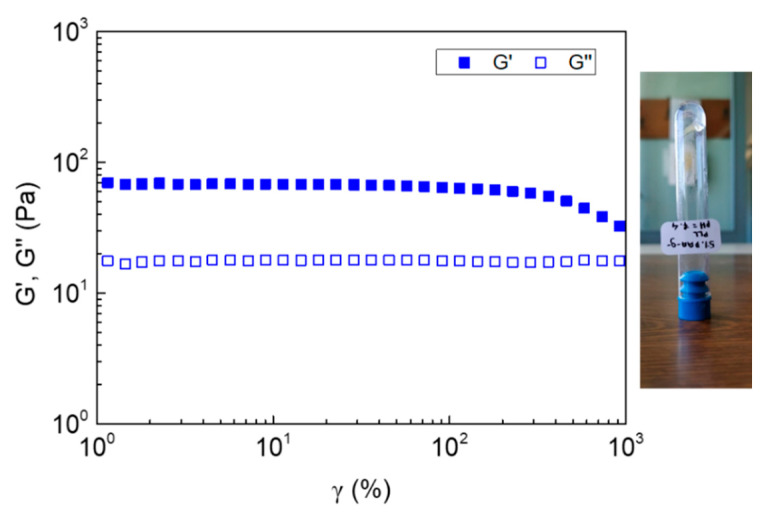
G’ and G” as a function of strain at 1 Hz of the PAA-g-PLL aqueous formulation (C_P_ = 5 wt %, pH 7.4). The digital photo depicts the free-standing hydrogel.

An interesting feature of this hydrogel is that it is easily hydrolysable in strongly acidic media. The labile boc groups protecting the amines of the PLL side groups are hydrolyzed instantly in the presence of excess TFA (very low pH), inducing network rupture given that P(b-LL) loses its hydrophobicity, which is responsible for copolymer association. However, the resulting PAA-g-PLL graft copolymer will re-associate at pHs in which both components are ionized (exhibiting oppositely charged moieties), through ionic interactions. We have observed that in the low concentration regime (C_P_ ≤ 1.2 wt%) of the PAA-g-P(b-LL) hydrogel, the PAA-g-PLL cannot form a percolated structure, thus exhibiting a viscous sol (see Figure S5 in Supplementary Materials). In Figure 10, a preliminary strain sweep experiment for a 5 wt% PAA-g-PLL shows gel-like properties (confirmed by the digital image) with a storage modulus of 67 Pa (1 Hz) and the linear viscoelastic regime expanding at a strain amplitude of about 200%. It is obvious that the two graft copolymers, having similar macromolecular architecture but different associative perspectives (hydrophobic versus ionic interactions), exhibit quite different gelation capabilities and properties. The hydrogels formed by PAA-g-PLL association are currently under investigation and the results will be reported in a forthcoming paper.

## 3. Conclusions

A novel amphiphilic graft copolymer consisting of a weak anionic polyelectrolyte (PAA) as a backbone and grafted by biodegradable poly(boc-L-lysine) was designed and explored in aqueous media as the gelator. PAA-g-P(b-LL) self-assembles spontaneously in aqueous media, triggered by the hydrophobic association of the pendant P(b-LL) (stickers) forming free-standing hydrogels at relatively low polymer concentrations (0.25–1.20 wt%). Oscillatory shear rheology was employed to estimate the rheological behavior of the hydrogel. The rheological analysis demonstrated that the copolymer behaves as a strong, elastic, soft material in aqueous media, showing characteristics of a “frozen” network, thanks to the highly hydrophobic nature of the stickers. It is suggested that the hydrogel structure is constituted of densely packed swollen microgels as dictated by the easy liquification upon applying low shear (LVR < 1 %). Step-strain oscillatory shear measurements showed that the hydrogel flows instantly upon applying a low strain (induced by low stress) and instantly self-heals when the stress is relaxed. Moreover, step-wise shear rate steady state measurements showed that the viscosity under shear, when simulating injection through a 28-gauge syringe needle, can be regulated at lower than 1 Pa.s, affording effortless injectability. Remarkably, the hydrogel responds to pH changes due to the pH-dependence of the degree of ionization of PAA but is resistant to temperature variations thanks to the “frozen” physical cross-links. A feature of PAA-g-P(b-LL) copolymers is that they can be easily hydrolyzed to transform from an amphiphilic to a double hydrophilic copolymer (PAA-g-PLL) bearing oppositely charged functions (-COO^−^, -NH_3_^+^), associative through ionic interactions, forming hydrogels with quite different properties.

Overall, the PAA-g-P(b-LL) aqueous formulations exhibit remarkable features as tailor-made injectable hydrogels. Shear responsiveness, pH-sensitivity and polymer concentration allow the design of the hydrogel properties, e.g., elasticity, injectability, self-healing, etc. Thanks to the biocompatibility of both constituents, the graft copolymer and the additional biodegradability of the P(b-LL) grafts, the afforded hydrogels could be tailored to target various potential biomedical applications such as controlled drug delivery, tissue engineering, wound healing, etc. Given that the rheological behavior seems similar to Carbopol-based hydrogels (chemically cross-linked PAA microgels) which have found a plethora of pharmaceutical applications [31], the present PAA-g-P(b-LL)-based material, incorporating biodegradable cross-links, could be considered for advanced applications.

## 4. Materials and Methods

### 4.1. Materials

Boc-PLL polypeptide was synthesized by ring opening polymerization of the ε-tert-butyloxycarbonyl-L-lysine-N-carboxy anhydride (NCA), with the following molecular weight characteristics: Mw= 11,980 Daltons, Mn = 11,365 Daltons (degree of polymerization 50) and molecular polydispersity, Mw/Mn, = 1.054. Details are reported elsewhere [32]. PAA (Mw = 450,000 Daltons) was purchased from Polysciences (Warrington, USA). N, N′-Dicyclohexylcarbodiimide (DCC, Alfa Aesar, MA, USA), 1-Hydroxybenzotriazole hydrate (HOBt, Fluka, NC, USA) were used as received. Dimethylformamide (DMF, Aldrich, MA, USA), hydrochloric acid (HCl, Panreac, Chicago, IL, USA), sodium hydroxide (NaOH, Panreac, Chicago, IL, USA), diethyl ether (DEE), tetrahydrofuran (THF), methanol and trifluoroacetic acid (TFA) were purchased from Sigma-Aldrich (Athens, Greece) and used without further purification. Deuterated solvents deuterium oxide (D_2_O and deuterated dimethyl sulfoxide (DMSO-d_6_)) (from Sigma-Aldrich, St. Louis, MO, USA) were used as received. Ultra-pure 3-distilled water (3D-H_2_O) was obtained by means of an ELGA Medica R7/15 device.

### 4.2. Synthesis and Characterization of PAA-g-P(b-LL)

**Scheme 1:**
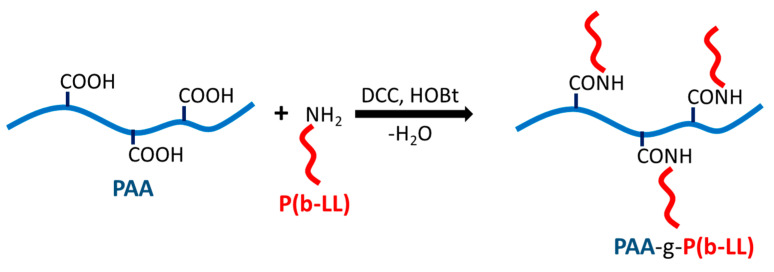
The grafting onto reaction for the synthesis of PAA-g-P(boc-LL). The free COOH groups along the PAA backbone of the graft copolymer have been omitted.

PAA-g-P(b-LL) was synthesized through the grafting onto method. In particular, carbodiimide chemistry was used for the reaction of the pendant carboxyl groups of the PAA (backbone) with the N-termini of P(b-LL) grafting chains forming amide bonds (Scheme 1) according to standard methods [33].

Specifically, amide bonds were formed by the reaction of the carboxyl group (-COOH) of PAA with the free α-amine group (-NH_2_) of protected P(boc-LL). The reaction was conducted under a nitrogen environment and at room temperature using DMF as solvent and DCC and HOBt as the coupling agents. HOBt was added in order to improve the reaction’s efficiency. Initially, 1.3 g (2.9 10^−6^ moles) of PAA, 0.4 g (3.5 10^−5^ moles) of P(boc-LL), 0.073 g (3.5 10^−4^ moles) of DCC (10 x moles of protected polypeptide P(boc-LL)) and 0.047 g (3.5 10^−4^ moles) of HOBt (10 x moles of protected polypeptide P(boc-LL)), were weighed and dissolved separately in 37 mL, 10 mL, 3 mL and 3 mL of DMF, accordingly, and left to stir for 24 h. When fully dissolved in DMF, the PAA solution was degassed with nitrogen for 15 min inside a 250 mL round bottom flask. Next, full homogenous solutions of DCC, HOBt and P(boc-LL) were degassed with nitrogen for few minutes and added successively into the PAA mixture. The reaction was left to proceed for 3 days at 25 °C under a nitrogen environment, using a refrigerated bath circulator.

**Figure 11:**
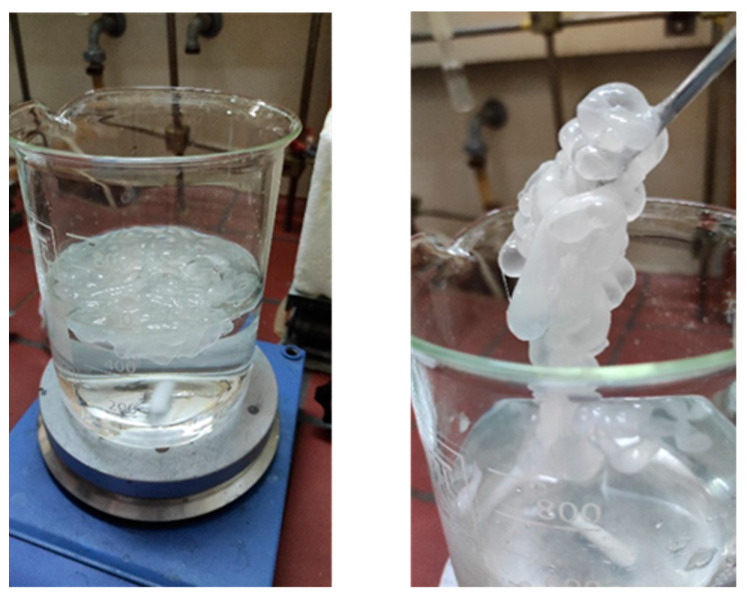
Images of jellification upon adding the reaction media in 1N NaOH.

To isolate the graft copolymer, the reaction media was treated with excess amount of 1N NaOH, upon which a polymeric gel was immediately formed during addition (see Figure 11). Afterwards, the gel was separated from the solution and the polymer was precipitated using methanol. The precipitate was rinsed several times with THF (anti-solvent of the copolymer) to remove possible organic impurities and unreacted P(b-LL). The final product PAA-g-P(b-LL) (1.58 g,) was obtained as white powder by drying in a vacuum oven.

The characterization of the graft copolymer in terms of grafting density (number of P(b-LL) per PAA backbone) was accomplished in the hydrolyzed counterpart (PAA-g-PLL) (see SI) by proton nuclear magnetic resonance spectroscopy (**^1^**H-NMR), using a BRUKER AVANCE III HD PRODIGY ASCEND TM 600 MHz spectrometer (Billerica, MA, USA) at 25 °C. The number of grafts per chain was determined to be about 11 side chains per backbone (see Supplementary Materials), which is very close to the target of 12 grafts per PAA (hence, the yield of the grafting reaction was about 90%).

### 4.3. Preparation of Samples for ^1^H-NMR Spectroscopy

The graft copolymer was first deprotected from the tert-butoxycarbonyl moieties (boc groups) of the protected PLL by acidic hydrolysis, according to the standard procedure (see Supplementary Materials). Afterwards, an aqueous solution (50 mL) of the copolymer was dialyzed against ultra-pure water using a dialysis membrane (MWCO: 12000 Da) for three days and the final PAA-g-PLL product was freeze-dried and collected from a vacuum drying oven. To determine the copolymer composition and the grafting density of the modified copolymer, PAA-g-PLL, the sample was dissolved in deuterated water (D_2_O). The ^1^H-NMR spectrum is presented in Figure S1 in Supplementary Materials.

### 4.4. Hydrogel Sample Preparation

Aqueous solutions of PAA-g-P(b-LL) graft copolymer were prepared under various pH conditions and different concentrations, as shown in Table 1. All the aqueous solutions were highly viscous. The samples were homogenized using a Vortex mixer machine and a Sigma 2K15 centrifugation machine (2000–5000 rpm and 25 °C) and left to fully dissolve on a lab shaker for one day. Purified 3-distilled water (3D-H2O) was used (ELGA MedicaR7/15 device) (ELGA Labwater, Woodridge, IL, USA). and the pH value of the system was adjusted by adding HCl or NaOH solution (1M).

### 4.5. Rheology

The rheological properties of the graft copolymer hydrogels were studied using a stress-controlled AR-2000ex (TA Instruments, New Castle, DE, USA) rheometer, with a cone and plate geometry (diameter 20 mm, angle 3°, truncation 111 μm), equipped with a Peltier plate system that allows the precise control of temperature (± 0.1 °C) and a solvent trap that prevents changes in concentration due to water evaporation. The experiments were performed at 25 °C. Several rheological experiments were carried out, namely an oscillation strain sweep at constant frequency of 1 Hz, an oscillation frequency sweep at strain amplitude (0.05%) in the linear viscoelastic regime, an oscillation temperature ramp (γ = 0.05%, 1 Hz), a stepwise oscillation time sweep at strains below and above the LVR limit (1 Hz) and a stepwise time sweep steady state flow at constant shear rates.

### 4.6. Scanning Electron Microscopy

The sample (C_P_ = 0.5 wt%, pH 7.4) was prepared in the same manner above-mentioned in Section 2.4. Afterwards, it was frozen in liquid nitrogen, then lyophilized and then the dried hydrogel was spattered with gold. SEM micrographs were obtained by a Bruker SEM, LEO-SUPRA VP35 with EDX Microanalysis Unit, at FORTH/ICE-HT in Patras, Greece.

## Figures and Tables

**Table 1:** Characteristics of the samples.

Sample	Concentration (wt%)	pH
S1	1.2	8.8
S2	1	8.8
S3	0.74	8.8
S4	0.5	8.8
S5	0.25	8.8
S6	0.5	7.4
S7	0.5	6
S8	0.5	5

## Data Availability

The data presented in this study are available on request from the corresponding author.

## Supplementary Materials

The following are available online at https://www.mdpi.com/article/10.3390/gels8120817/s1, Figure S1: ^1^H-NMR spectrum of PAA-g-PLL; Figure S2: Strain sweep of 1 wt % PAA-g-P(b-LL); Figure S3: Strain sweep of 5 wt % PAA-precursor; Figure S4: SEM images and pore size estimation of the freeze-dried hydrogel (0.5 wt % PAA-g-P(b-LL); Figure S5: Strain sweep of 5 wt % PAA-LL.
