# Supplementary material for: pH-Responsive, Thermo-Resistant Poly(Acrylic Acid)-g-Poly(boc-L-Lysine) Hydrogel with Shear-Induced Injectability
Source: Gels. 2022 Dec 12;8(12):817. doi: 10.3390/gels8120817 (PMC9778197; doi:10.3390/gels8120817)
Supplement: Supplementary file 1 [file gels-08-00817-s001.zip › gels-2097815-supplementary.pdf]

Supplementary Materials

pH-Responsive, Thermo-resistant Poly(Acrylic Acid)-g-Poly(boc-L-Lysine) Shear-Induced Injectable Hydrogel

M-H. Karga,[a] M-H. Kargaki,[a] H. Iatrou,[b] C. Tsitsilianis[a*]

[a] *Department of Chemical Engineering, University of Patras, 26500 Patras, Greece*

[b] *Department of Chemistry, University of Athens, Panepistimiopolis, Zografou, 15771 Athens, Greece*

A 0.3 wt% of aqueous solution of PAA-g-P(b-LL) was treated with a large excess of TFA (with respect of b-LL units) to deprotect the L-Lysine residues of the grafting chains. Upon adding the acid, the highly viscous solution (gel) turned immediately to sol confirming the hydrolysis of the hydrophobic boc moieties. Then the pH of the solution was adjusted to neutral by adding K_2CO_3 (1M). The solution then was dialyzed against ultra-pure water using a dialysis membrane (MWCO 12000 Da) for three days and the final PAA-g-PLL product was freeze dried and collected from a vacuum drying oven.

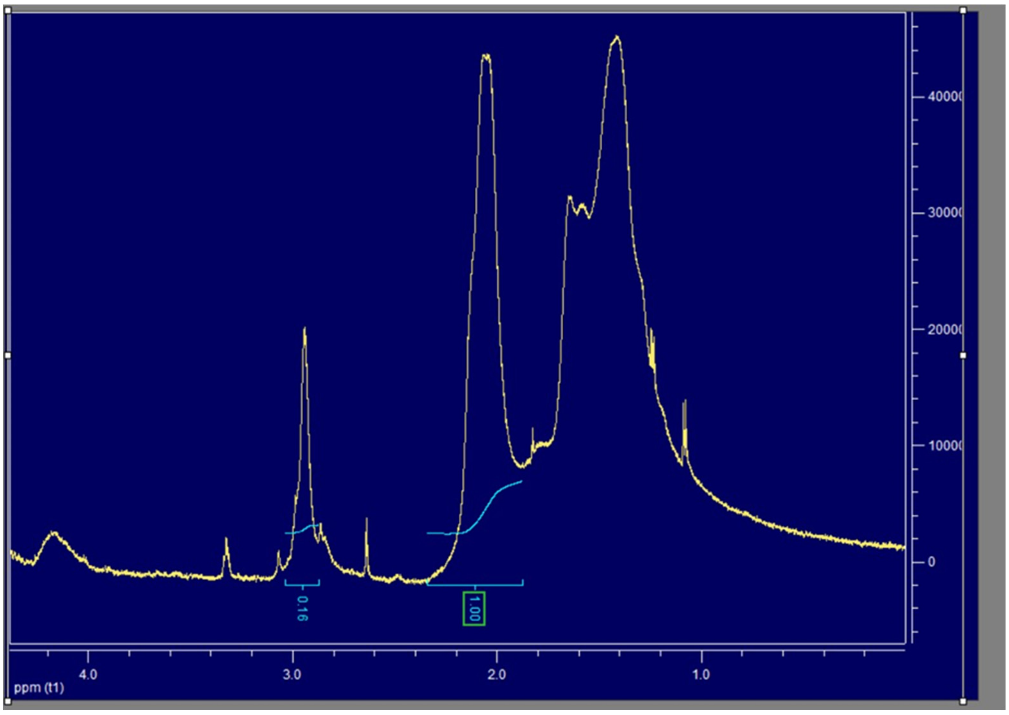

Figure S1. ^{1}H NMR spectra of PAA-g-PLL.

The composition of PAA-g-PLL after deprotection of boc-LL was determined by the ^{1}H NMR spectra of Figure S1, obtained in deuterated water (D_2O) using the characteristic peak of the (-C**H**-CH_2, 2.1 ppm) proton of the acrylic acid monomer repeating units of the PAA backbone chain and the (-C**H_2**, ~2.9 ppm) two protons next to the primary amino group of PLL lysine monomers. Hence, the graft copolymer comprises 8 mol % L-Lysine residues which corresponds to about 11 PLL or P(b-LL) grafts per PAA, taking into account that the mean degree of polymerization of PAA and PLL are 6250 and 50 respectively.

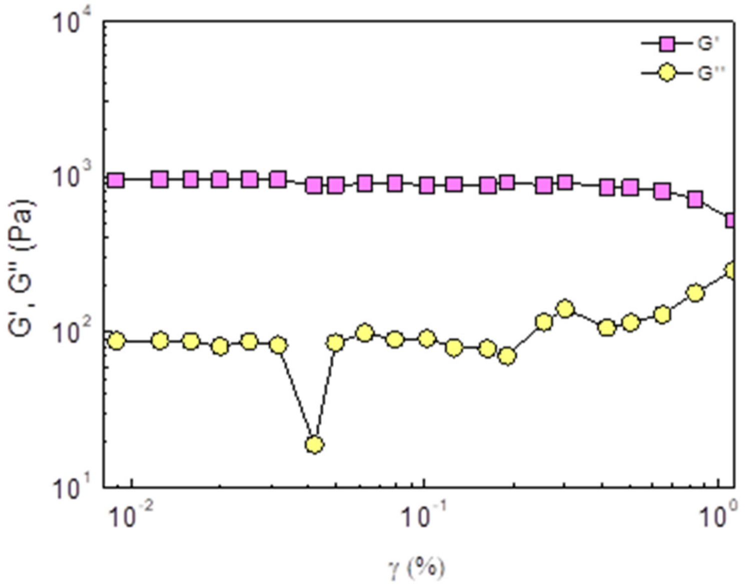

Figure S2. Strain sweep at 1 Hz of 1 wt % PAA-g-P(b-LL) aqueous formulation (pH 8.8).

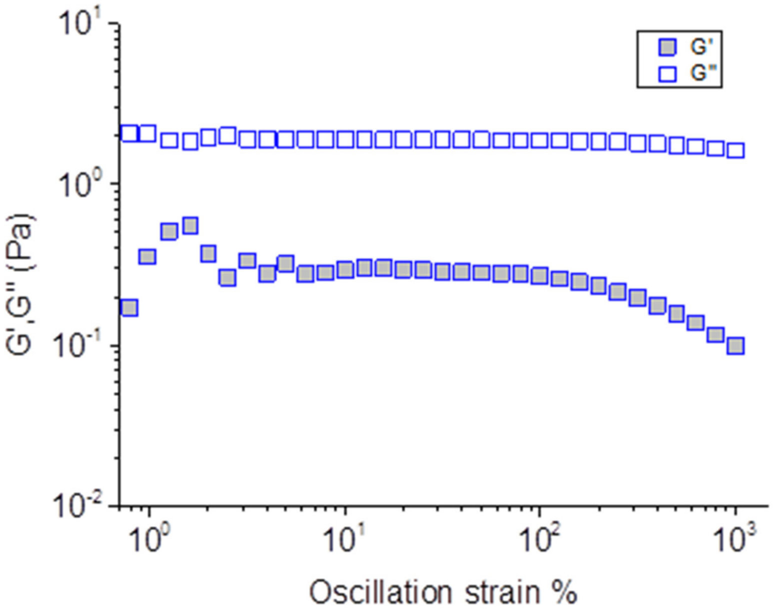

Figure S3. Strain sweep at 1 Hz of 5 wt % PAA-precursor aqueous formulation (pH 8.8).

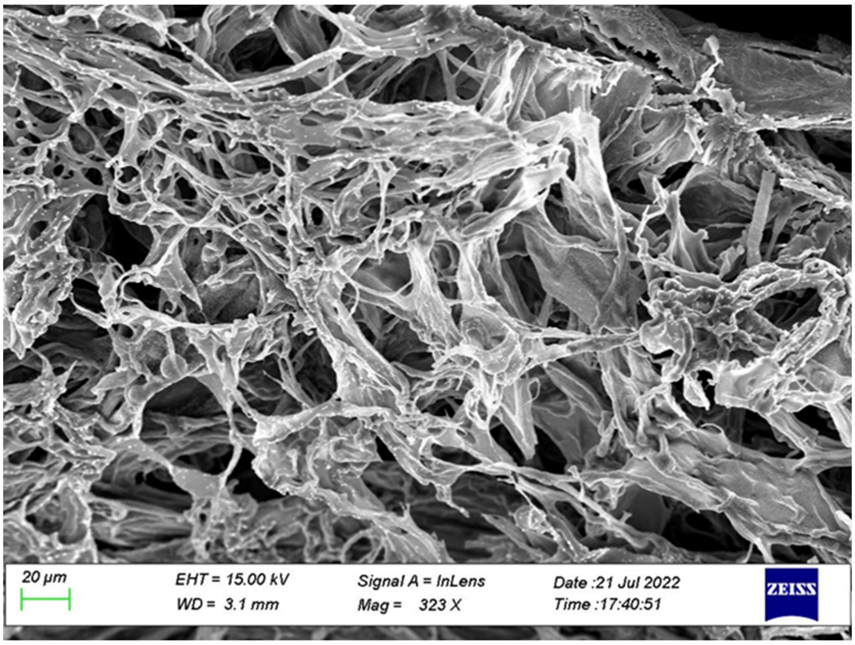

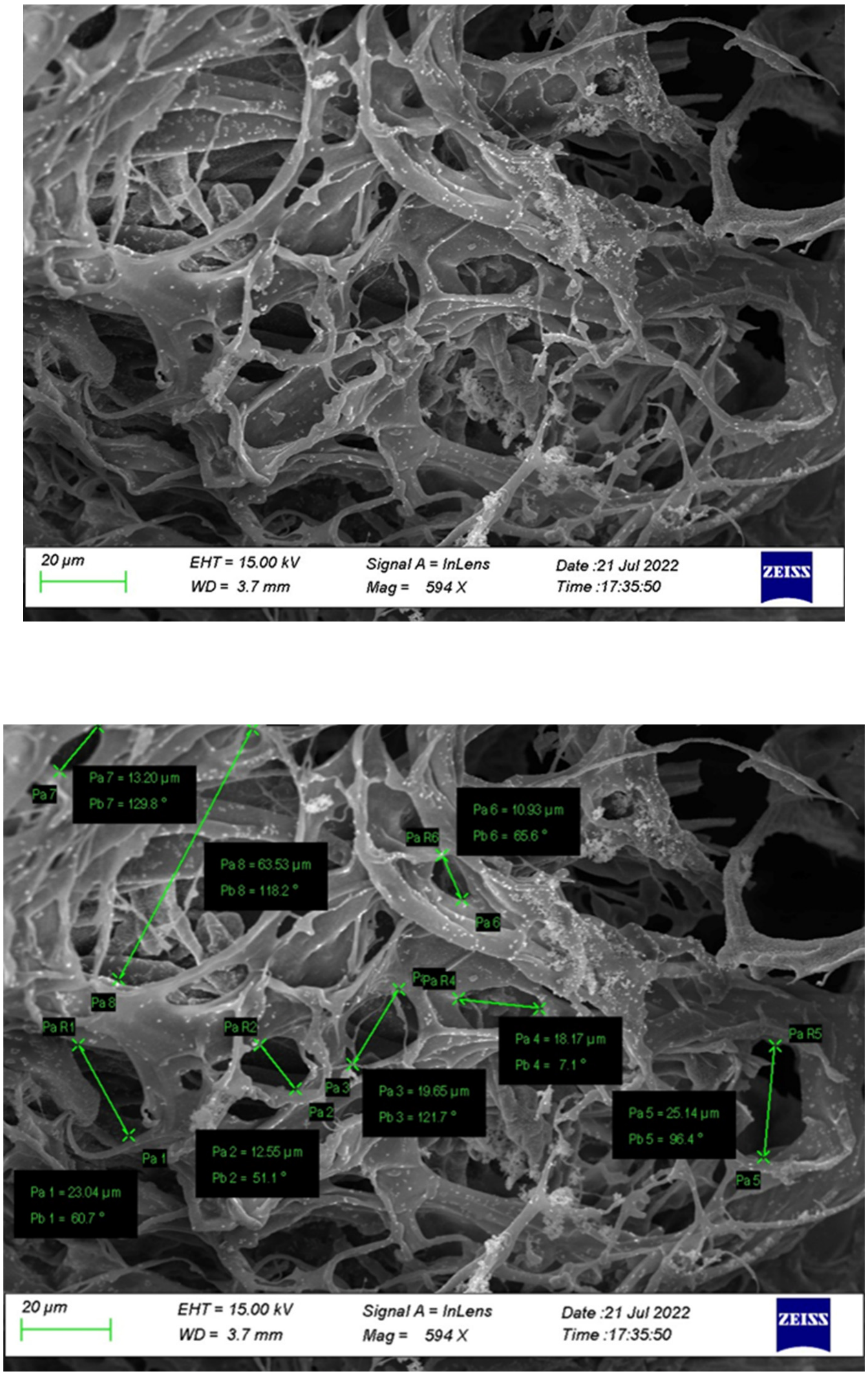

Figure S4. SEM images and pore size estimation of the freeze-dried hydrogel (0.5 wt % PAA-g-P(b-LL).

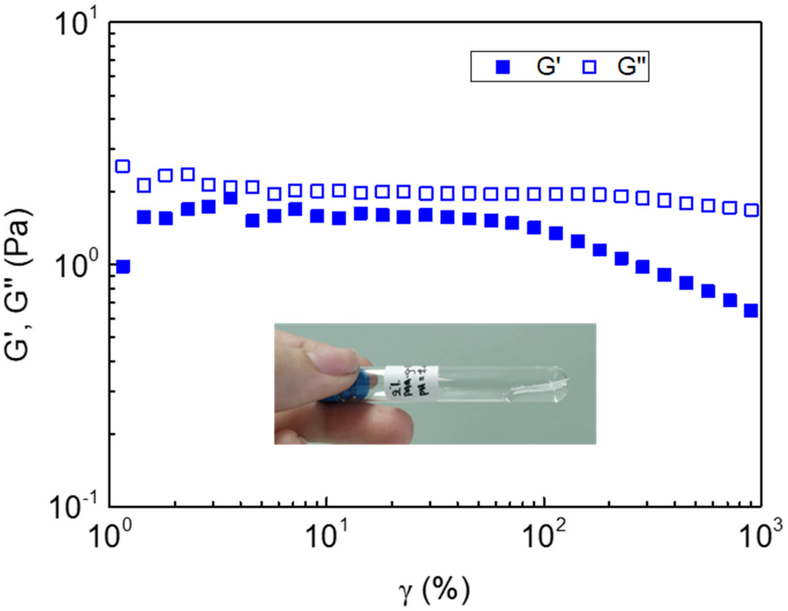

Figure S5. G’, G” as a function of strain at 1 Hz of the PAA-g-PLL aqueous formulation (C_P = 2 wt %, pH 7.4) showing liquid like behavior (inset).
